# Angiogenin Enhances Cell Migration by Regulating Stress Fiber Assembly and Focal Adhesion Dynamics

**DOI:** 10.1371/journal.pone.0028797

**Published:** 2011-12-14

**Authors:** Saisai Wei, Xiangwei Gao, Juan Du, Jinfeng Su, Zhengping Xu

**Affiliations:** 1 Institute of Environmental Medicine, Zhejiang University School of Medicine, Hangzhou, China; 2 College of Life Science, Zhejiang University, Hangzhou, China; Ottawa Hospital Research Institute, Canada

## Abstract

Angiogenin (ANG) acts on both vascular endothelial cells and cancer cells, but the underlying mechanism remains elusive. In this study, we carried out a co-immunoprecipitation assay in HeLa cells and identified 14 potential ANG-interacting proteins. Among these proteins, β-actin, α-actinin 4, and non-muscle myosin heavy chain 9 are stress fiber components and involved in cytoskeleton organization and movement, which prompted us to investigate the mechanism of action of ANG in cell migration. Upon confirmation of the interactions between ANG and the three proteins, further studies revealed that ANG co-localized with β-actin and α-actinin 4 at the leading edge of migrating cells. Down-regulation of ANG resulted in fewer but thicker stress fibers with less dynamics, which was associated with the enlargements of focal adhesions. The focal adhesion kinase activity and cell migration capacity were significantly decreased in ANG-deficient cells. Taken together, our data demonstrated that the existence of ANG in the cytoplasm optimizes stress fiber assembly and focal adhesion formation to accommodate cell migration. The finding that ANG promoted cancer cell migration might provide new clues for tumor metastasis research.

## Introduction

Angiogenin (ANG) is up-regulated in various types of human cancer, including breast, cervical, colon, colorectal, endometrial, gastric, liver, kidney, ovarian, pancreatic, prostate, and urothelial cancers, as well as astrocytoma, leukemia, lymphoma, melanoma, osteosarcoma, and Wilms' tumor [1], indicating a close relationship between ANG and tumor development. Traditionally, ANG has been recognized as an angiogenic factor which promotes angiogenesis by activating endothelial and smooth muscle cells and inducing the formation of tubular structures [2]–[4]. Recently, ANG has been reported to directly enhance the proliferation of cancer cells such as HeLa cells and PC-3 cells, indicating that ANG plays dual roles in cancer progression by acting on both vascular and cancer cells [1], [2], [5], [6].

ANG exerts its functions both extracellularly and intracellularly. Extracellular ANG activates signal-related kinase1/2 (ERK1/2) in human umbilical vein endothelial cells (HUVECs) or stress-associated protein kinase/c-Jun N-terminal kinase (SAPK/JNK) in human umbilical artery smooth muscle cells (HuASMCs) [3], [4]. Meanwhile, ANG can be internalized and translocated to the nucleolus where it enhances rRNA transcription and ribosome biogenesis to meet the high demand for protein synthesis during cell proliferation [7]. Evidence shows that ANG also localizes in the cytoplasm [1], [8], [9], but the role of the cytosolic ANG is largely unknown.

ANG has been reported to promote endothelial cell migration [10], [11]. Cell migration is a highly complex and regulated process which requires the integrated activities of cytoskeleton reorganization and cell-matrix interaction. During migration, cells attach to the matrix *via* focal adhesions (FAs) [12], while stress fibers anchor to FAs at their ends and generate forces to move and reshape the cell [13]. The assembly and disassembly of FAs, and the movement of stress fibers coordinately lead the cells to migrate [14]. It was reported that the secreted ANG attaches to the extracellular matrix (ECM) and serves as a substratum to facilitate endothelial cell adhesion and spreading [15], [16]. ANG binds to a smooth muscle type α-actin on the endothelial cell surface [17], and the subsequently dissociated ANG-actin complex promotes the degradation of the basement membrane to enhance cell invasion and migration [10]. On the other hand, ANG activates the protein kinase B/Akt signaling pathway to promote HUVEC migration [11]. A recent study showed that ANG inhibits actin polymerization at sub-physiological KCl concentrations *in vitro* [18], suggesting that ANG influences cytoskeletal organization directly. However, the precise role of ANG in cytoskeletal organization and cell migration remains to be elucidated.

To better understand the intracellular roles of ANG, we have performed a co-immunoprecipitation coupled mass spectrometry (MS) analysis to identify potential ANG-interacting proteins. Among the obtained 14 candidate ANG-binding proteins, β-actin, α-actinin 4, and non-muscle myosin heavy chain 9 are stress fiber components. After confirmation of the interactions between ANG and the three proteins, we explored the biological role of ANG in stress fiber formation, focal adhesion dynamics, and cell migration.

## Results

### Identification and functional classification of ANG-interacting proteins

**Figure 1:**
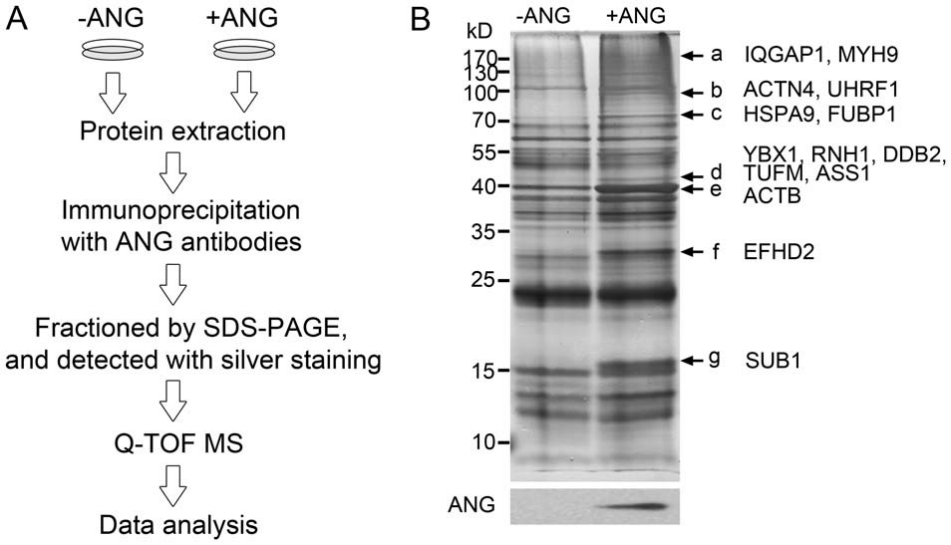
Identification of ANG-associated proteins. (A) Schematic illustration of the strategy used to screen ANG-associated proteins. (B) Proteins immunoprecipitated with anti-ANG antibodies from total lysates of HeLa cells with (+ANG) or without (−ANG) ANG were fractionated by 12% SDS-PAGE gel. The gels were either visualized by silver staining (upper panel) or blotted with anti-ANG antibodies (lower panel). The differential bands (marked a–g) were subjected to trypsin digestion and Q-TOF MS analysis. The identified proteins were listed along with the corresponding bands.

To screen potential ANG-interacting proteins, we used a co-immunoprecipitation combined with MS approach. The extracellular ANG can be internalized by its target cells such as HeLa cells and human umbilical vein endothelial cells (HUVECs) [5], [7]–[9], [19], [20], possibly through an endocytosis pathway [19]. After treating the HeLa cells with exogenous ANG, the intracellular level of this protein increased (Figure 1B lower panel). Accordingly, the immunoprecipitated complex from exogenous ANG-treated cells contained more ANG-interacting proteins than that from the untreated HeLa cells, shown as enhanced bands in silver-staining gel (Figure 1B, upper panel). Therefore, seven obviously enhanced bands were subjected to protein identification by MS (Figure 1B marked a–g). The MS data were applied to NCBI database searching. Three types of protein were filtered out during the analysis: keratins; proteins in both the control (ANG−) and ANG-treated (ANG+) groups (considered to be non-specifically trapped by the protein A agarose beads); and proteins that did not contain any peptides with >95% confidence. After organizing the data, we finally identified 14 putative ANG-associated proteins (Figure 1B, Table 1).

**Table 1:** List of potential ANG-interacting proteins identified by Q-TOF-MS analysis.

In-gel digestion*	Protein Name	Official Symbol	Accession no.	No. of peptides	% Cov(95)
a	Ras GTPase-activating-like protein IQGAP1	IQGAP1	gi|1170586	15	10.0
	Myosin-9	MYH9	gi|6166599	21	14.7
b	Alpha-actinin-4	ACTN4	gi|13123943	16	20.0
	E3 ubiquitin-protein ligase UHRF1	UHRF1	gi|67462077	2	2.8
c	Heat shock 70 kDa protein 9	HSPA9	gi|21264428	3	5.4
	Far upstream element-binding protein 1	FUBP1	gi|116241370	3	6.5
d	Y-box-binding protein 1	YBX1	gi|116283293	2	10.1
	Ribonuclease/angiogenin inhibitor 1	RNH1	gi|132573	7	20.8
	DNA damage-binding protein 2	DDB2	gi|12230033	4	13.2
	Elongation factor Tu, mitochondrial	TUFM	gi|1706611	1	2.7
	Argininosuccinate synthetase	ASS1	gi|20141195	2	5.3
e	Actin, beta	ACTB	gi|16359158	9	24.5
f	EF-hand domain-containing protein D2	EFHD2	gi|20140139	5	27.5
g	activated RNA polymerase II transcription cofactor 4	SUB1	gi|19923784	2	14.9

*a–g: refer to Figure 1 B.

To understand the functions of the potential ANG-interacting proteins, we carried out function annotations which have been provided by the Database for Annotation, Visualization, and Integrated Discovery (DAVID, http://david.abcc.ncifcrf.gov/). The annotation revealed that the proteins are localized in the cytoplasm or the nucleus, and are involved in various biological processes, including transcription (4 proteins), duplication (2), regulation of actin cytoskeleton (4), cell migration (3), and cell adhesion (4) (Table 2). Among the annotated functions, cell migration, cell adhesion, and regulation of actin cytoskeleton are closely related processes. The stress fiber components β-actin (ACTB), α-actinin 4 (ACTN4), and non-muscle myosin heavy chain 9 (MYH9) were classified as being involved in all these functions (Table 2). Moreover, the three proteins were of high peptide coverage (24.5, 20.0, and 14.7%) (Table 1), indicating the high content of these proteins in the precipitates. Therefore, we selected ACTB, ACTN4 and MYH9 for further investigation.

**Table 2:** Function annotations of the 14 putative ANG-binding proteins by DAVID.

Biological process	Candidate proteins
Transcription	FUBP1, UHRF1, YBX1, SUB1
Duplication	IQGAP1, RNH1
Regulation of actin cytoskeleton	IQGAP1, MYH9, ACTN4, ACTB
Cell adhesion	IQGAP1, MYH9, ACTN4, ACTB
Cell migration	MYH9, ACTN4, ACTB
Proteolysis	MYH9, UHRF1, DDB2,
Protein transport	ACTN4, HSPA9, MYH9
Other functions	ASS1,EFHD2, TUFM

### ANG interacts with stress fiber components at the leading edge of migrating cells

**Figure 2:**
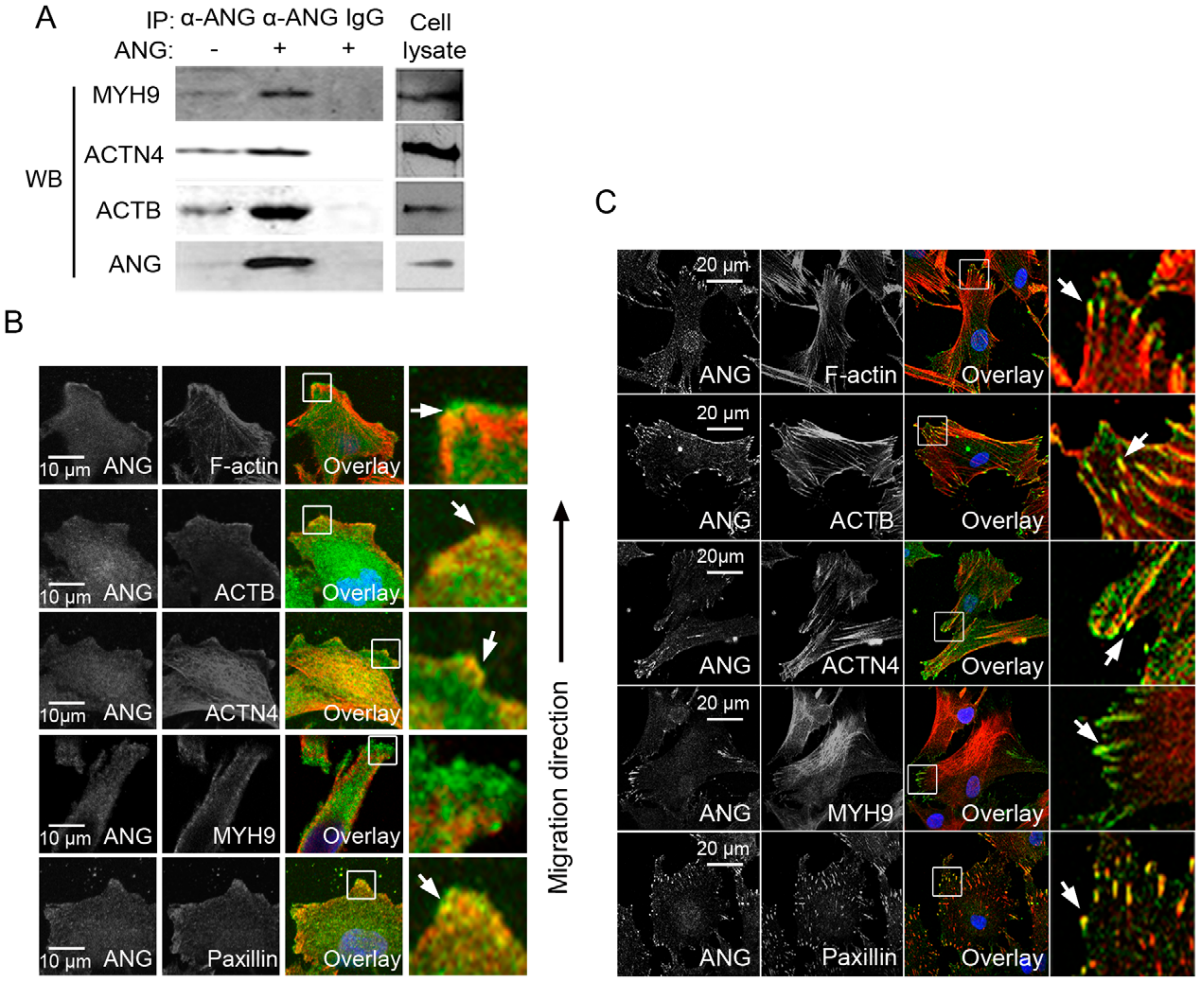
ANG interacted with stress fiber components at the leading edge of migrating cells. (A) HeLa cells were treated with or without ANG, and the lysates were immunoprecipitated with goat anti-ANG antibodies or goat IgG. The immunoprecipitates and whole cell lysate (Cell lysate) were fractionated by SDS-PAGE and blotted with anti-MYH9, anti-ACTN4, anti-ACTB, or anti-ANG antibody. HeLa cells (B) and HSF cells (C) were fixed and double-stained for ANG (green) and F-actin, ACTB, ACTN4, MYH9 or paxillin (red). The merged images (overlay) and the magnified views are shown in the right panel. The white arrows indicate the overlapped sites.

To confirm the interactions between ANG and ACTB, ACTN4, or MYH9, we performed independent precipitations with an anti-ANG antibody or normal IgG. The results revealed that the three proteins could be pulled down with endogenous ANG (ANG−, Figure 2A). The intracellular level of ANG increased when HeLa cells were treated with exogenous ANG (ANG+, Figure 2A), which is consistent with the previous report that HeLa cells can uptake this protein [5]. The co-precipitated amounts of ACTB, ACTN4, and MYH9 also increased (ANG+, Figure 2A), further demonstrating the authenticity of these interactions. It was worth to point out that the expression levels of these three ANG-interacting proteins did not change in response to ANG treatment (Figure S1).

**Figure 3:**
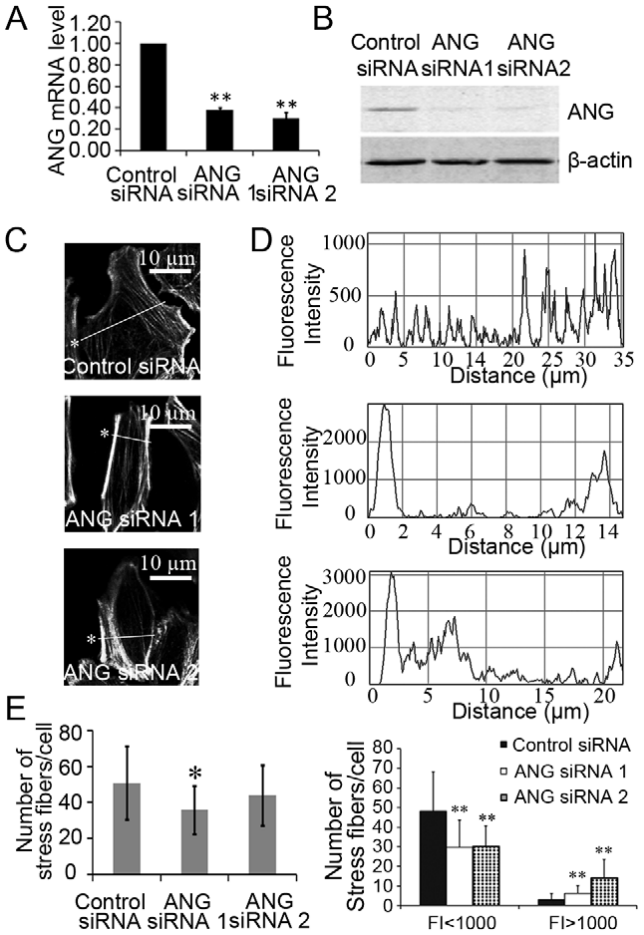
Down-regulation of ANG increased the assembly of stress fibers. HeLa cells were treated with ANG siRNAs or control siRNA. ANG mRNA levels (A) and protein levels (B) were detected. Similar treated cells were fixed and stained with rhodamine-phalloidin (C). Scale bar: 10 µm. (D) Quantification of fluorescence intensity across the lines of the corresponding cells in (C) using ImageJ software. Asterisks demarcate cells quantified in (C) and their corresponding line graphs in (D). (E) Numbers of fluorescence intensity peaks of stress fibers in control and ANG knockdown cells. Fluorescence intensity (FI) was classified as low (FI<1000) or high (FI≥1000) in the right panel. **P<0.01.

We then explored the locations of these interactions in migrating cells using immunofluorescence analysis. HeLa cells in the wound healing assay were stained with anti-ANG monoclonal antibody, together with antibodies targeting ACTB, ACTN4, and MYH9. Rhodamine-phalloidin was used to mark F-actin. The data showed that the cytosolic ANG co-localized with ACTB and ACTN4 well at the leading edge, however, there was no obvious overlapping between ANG and MYH9 (white arrows, Figure 2B, C). Human skin fibroblast (HSF) cells also express endogenous ANG (Figure 3C, left panel), and we observed even clearer co-localization between ACTB, ACTN4, MYH9 and ANG (white arrows, Figure 3C). ANG also co-localized with the focal adhesion marker paxillin in both HeLa and HSF cells (Figure 2B, C), indicating that ANG and the stress fiber components form protein complexes at focal adhesions.

### Reduction of ANG enhances stress fiber assembly and reduces cytoskeleton dynamics

The interactions between ANG and the stress fiber components suggest that ANG regulates the organization of actin cytoskeleton. To test this hypothesis, we compared the distribution of F-actin in control and ANG-deficient cells. Quantitative RT-PCR and immunoblot analysis confirmed that small interfering RNAs targeting to ANG (ANG siRNA 1 and ANG siRNA 2) were capable of down-regulating ANG expression at both the mRNA and protein levels (Figure 3A,B). Consequently, fewer but larger stress fibers were observed in ANG-deficient cells as shown by rhodamine-phalloidin staining (Figure 3C). To quantify the stress fiber density in the cells, we incorporated a line profile across the cytoplasm using ImageJ software [21], which identified stress fibers by their increased fluorescence relative to areas devoid of stress fibers (Figure 3C). Sharp, distinct peaks in fluorescence intensity (FI) represented individual stress fibers crossed by the lines, and the width of the peak indicated the thickness of a stress fiber (Figure 3D). For statistical purposes, FI 1,000 was arbitrarily set to discriminate stress fiber as strong (FI>1000) or weak (FI<1000). The data revealed that the total number of stress fibers in ANG-deficient cells decreased (Figure 3E, left panel), but the number of strong stress fibers significantly increased, and the weak decreased (Figure 3E, right panel), demonstrating that ANG prevents stress fibers from over-assembly. A similar phenomenon was observed in HeLa cells stably transfected with an ANG interference plasmid (Figure S2).

**Figure 4:**
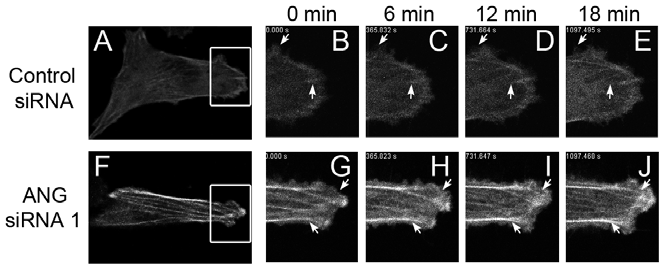
Down-regulation of ANG decreased the cytoskeleton dynamics in HeLa cells. HeLa cells were transfected with RFP-actin plus control siRNA or ANG siRNA. Fluorescence images were then taken at 2-min intervals for 18 min, and the images at 0 min, 6 min, 12 min and 18 min were shown. To qualitatively compare the position change of actin structure, two arrows were set at the fixed position in each set of images as a reference position. The upper arrow indicates growing pseudopodia, and the lower arrow points to stress fiber. (A) Representative fluorescence image of a HeLa cell transfected with RFP-actin and control siRNA. The images B–E magnify the boxed region in A at different time points. (F) Representative fluorescence image of a HeLa cell transfected with RFP-actin and ANG siRNA. The images G-J magnify the boxed region in F at different time points.

To gain further insight into the effects of ANG on cytoskeleton dynamics, we expressed the red fluorescent protein (RFP)-tagged actin, which permits the visualization of stress fibers in living cells. HeLa cells were transfected with plasmids encoding RFP-actin together with ANG siRNAs, or with control siRNAs, and viewed under a time-lapse confocal microscope. The images were taken every 2 min for 18 min. Similar to immunofluorescence results, down-regulation of ANG increased the density of the stress fibers (Figure 4A,F). To judge the movements of the actin structures, we set two fixed arrows on the serial images, which allowed to compare the positions of actin structures to these reference arrows. The lower arrow at each set of images was used for the judgement of stress fiber dynamics, while the upper one was for the pseudopodia movement. The data showed that the stress fibers within control cells changed positions rapidly as the cells migrated (lower arrow, Figure 4B–E & movie S1A). The stress fibers shown at 0 min disappeared and new stress fibers structure appeared at the right side of the arrow at 18 min. The pseudopodia around the cell periphery also moved and the new ones grew as time passed (upper arrow, Figure 4B–E & movie S1A). By contrast, the stress fibers in ANG-deficient cells were relatively static. Although its pseudopodia moved rapidly (upper arrow, Figure 4G–J & movie S1B), the inner network of actin cytoskeleton appeared virtually fixed in place (lower arrow, Figure 4G–J & movie S1B), indicating that ANG regulates stress fiber dynamics rather than pseudopodia.

### Reduction of ANG enlarges focal adhesions and blocks focal adhesion kinase activation

**Figure 5:**
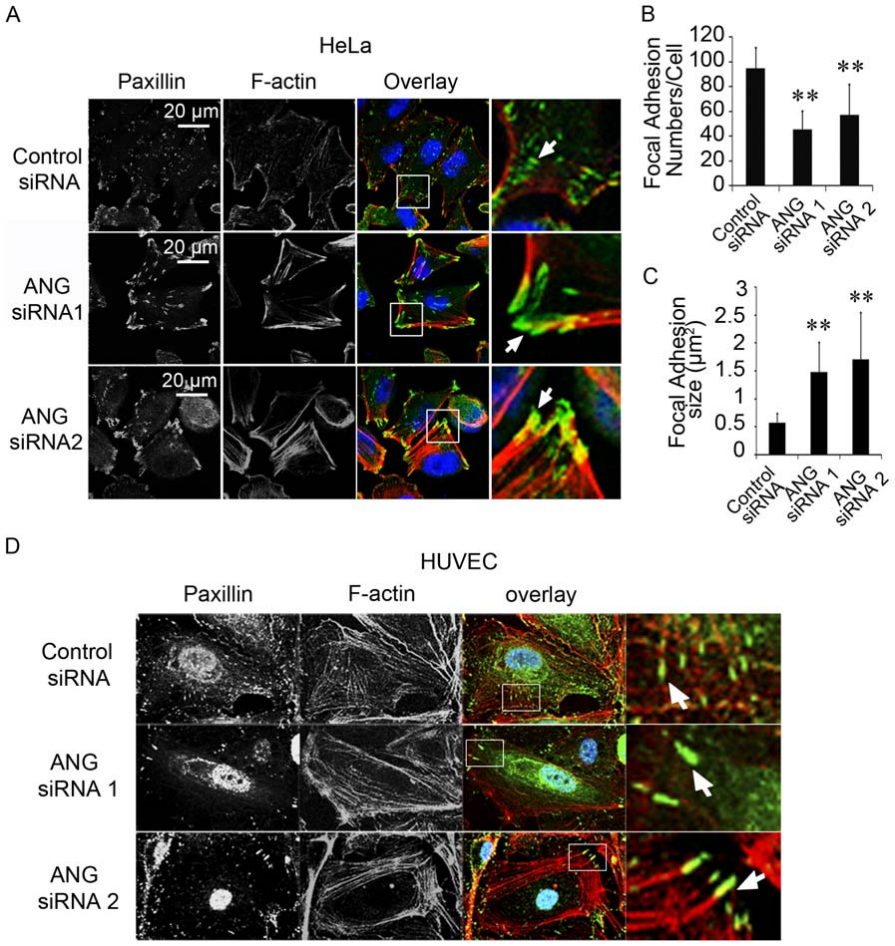
Down-regulation of ANG increased the formation of focal adhesions. (A) HeLa cells were treated with control siRNA, ANG siRNA 1 or ANG siRNA 2 for 48 h, then fixed and stained with rhodamine-phalloidin (red) and anti-paxillin antibody (green). Nuclei stained with DAPI (blue). (B) Numbers of paxillin-staining focal adhesions was quantified for control and ANG knockdown cells using ImageJ software. Seven cells were analyzed per condition in each experiment. Three independent experiments were performed. **P<0.01. (C) Average size of paxillin-containing focal adhesions in control and ANG-deficient cells. **P<0.01. (D) HUVECs were treated with control siRNA or ANG siRNAs, fixed, and stained with rhodamine-phalloidin (red) and anti-paxillin antibody (green). Nuclei were stained with DAPI (blue).

Stress fibers are connected to the substrate *via* FAs, and the changes in stress fibers often influence the FA formation [13]. FAs are large macromolecular assemblies that include integrin, focal adhesion kinase (FAK), paxillin, and vinculin [12]. Therefore, we checked the formation of FAs in ANG down-regulated cells using paxillin as a marker. The data revealed that the FAs were larger in ANG-deficient cells than in the control cells (Figure 5A). Analysis using ImageJ software [22] showed that the number of FAs decreased (Figure 5B), while the average area of FAs increased when ANG was down-regulated (Figure 5C). The data indicated that ANG prevents the over-assembly of focal adhesions.

**Figure 6:**
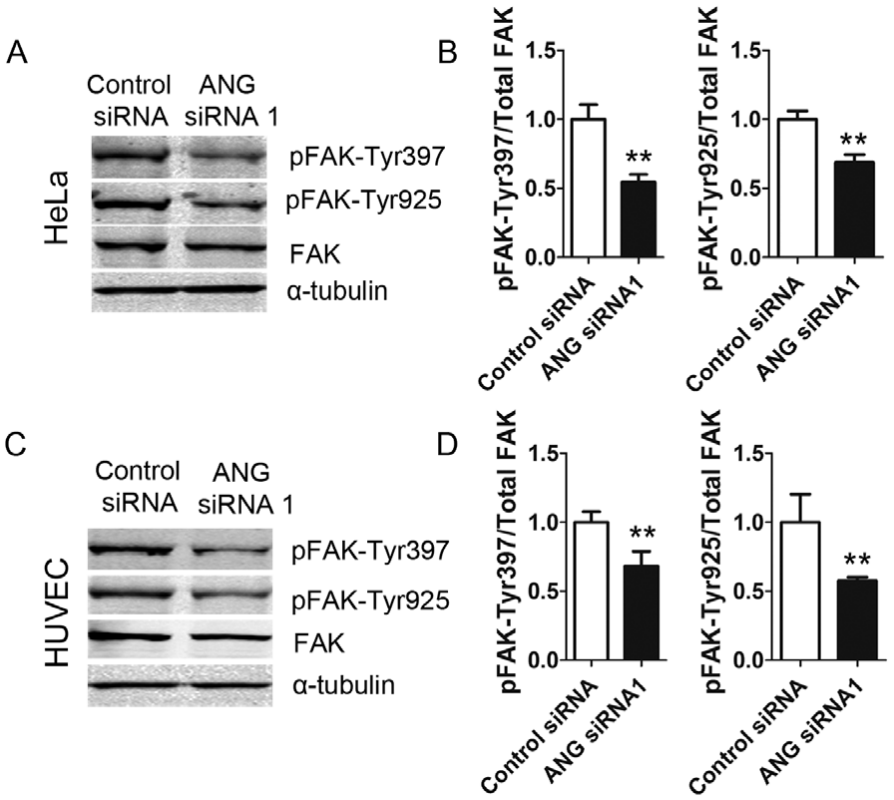
Down-regulation of ANG decreased FAK phosphorylation. HeLa cells (A) or HUVECs (C) were treated with ANG siRNA 1 or control siRNA. The cell lysates were subjected to immunoblot detection of pFAK-Tyr397, pFAK-Tyr925, FAK and α-tubulin. The densities of phosphorylated FAK bands were analyzed with Quantity One software and normalized to total FAK (B-HeLa cells, D-HUVECs). Data shown are the mean ± SD of three independent experiments.

FA enlargement at the cell periphery is a sign of defects in the FA dynamics, which is essential for cell movement [23]. Focal adhesion kinase (FAK) activity is a key factor in controlling FA dynamics [24]. Because the enlargements of FAs had been observed in ANG-deficient cells, we further measured the phosphorylations of FAK at Tyr-397 and Tyr-925, two main phosphorylation sites during FAK activation.. The results showed that downregulation of ANG decreased phosphorylations of FAK at Tyr-397 and Tyr-925 (Figure 6A, B), suggesting that ANG is essential for FAK activation and FA dynamics.

HUVEC is also a target cell of ANG, and its migration is prompted by ANG. Therefore, we further detected the effects of ANG on stress fiber formation, and focal adhesion formation in this type of cell. Data showed that the density of stress fibers and the size of FAs were increased in ANG-deficient cells (Figure 5D), and the phosphorylations of FAK at Tyr-397 and Tyr-925 were accordingly decreased (Figure 6C,D).

### Reduction of ANG attenuates cell migration

**Figure 7:**
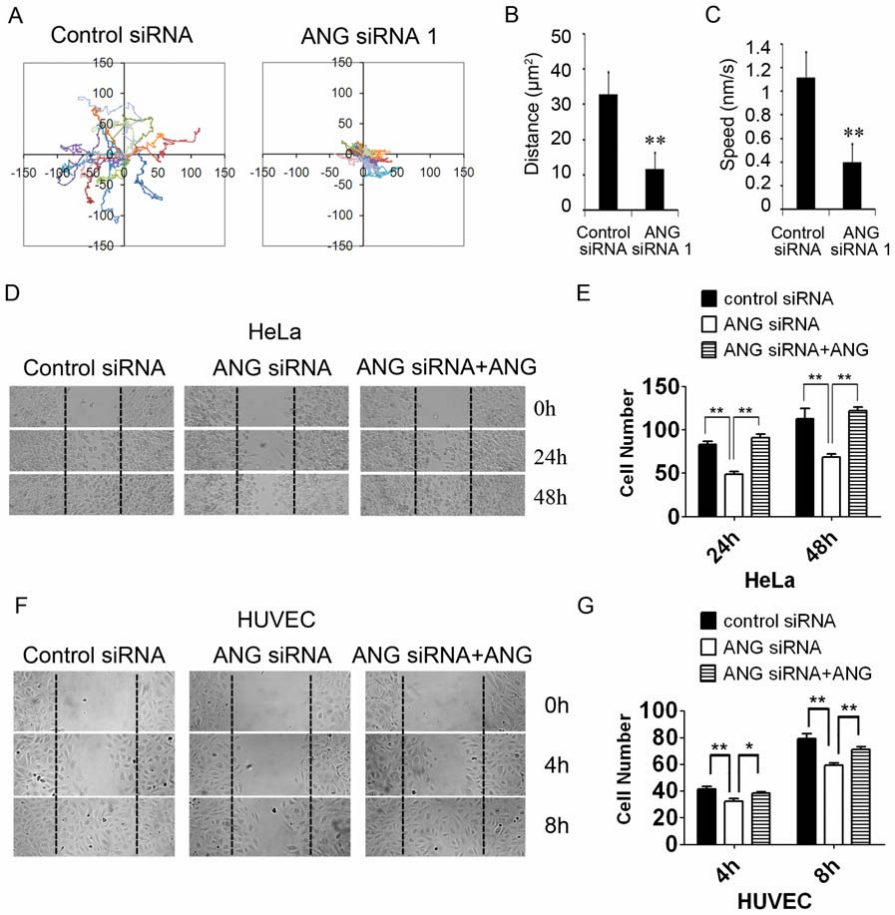
ANG deficiency attenuated cell migration. (A) HeLa cells were treated with ANG siRNA 1 or control siRNA. Movements of individual HeLa cells were traced by videomicroscopy. Migration tracks of 15 cells for each group are shown as scatter plots. The moving distance (B) and speed (C) of the tracked cells were calculated based on the data from each group provided by the ImageJ manual tracking plug-in. **P<0.01. HeLa cells (D) and HUVECs (F) were tranfected with ANG siRNA 1, treated with or without exogenous ANG, subjected to wound-healing assay, and then imaged at the indicated time. Cells transfected with control siRNA were used as control. Cells that migrated into the wound areas were counted and analyzed (E, G). Data shown are the mean ± SD of three independent experiments.

The above results strongly suggested a vital role of ANG in cell migration. Therefore, we further assessed the effect of ANG on HeLa cell migration using a time-lapse videomicroscope, which permits monitoring the migration of individual cells. Knockdown of ANG decreased the motility of HeLa cells (Figure 7A). Quantification of these movements revealed a dramatic reduction in moving distance and average speed in ANG-deficient cells compared to that in control cells (Figure 7B,C).

The wound healing assay showed similar results. The control cells migrated and closed the wound area within 48 h in HeLa cells and 8 h in HUVECs, while ANG siRNA-treated cells were significantly less motile (Figure 7D–G). Addition of exogenous ANG reversed the reduced cell migration capacity caused by ANG RNAi in both HeLa cells and HUVECs (Figure 7D–G), demonstrating that it is ANG that plays a regulatory role on cell migration.

## Discussion

To explore the role of cytosolic ANG, we performed a co-immunoprecipitation assay and identified 14 ANG-associated proteins. Function annotation classified ACTB, ACTN4, and MYH9, the three major stress fiber components, involve in cytoskeleton regulation, cell adhesion, and cell migration. Further studies revealed that the existence of ANG ensures optimized stress fiber assembly and focal adhesion formation, thus promotes endothelial and cancer cell migration.

Among the 14 candidate molecules, ribonuclease/angiogenin inhibitor 1 (RNH1) has already been reported to interact with ANG [25]. Interestingly, four of them function in transcription, showing that ANG might act as a transcription factor [5], [7], [26]. Two proteins were classified in the category of DNA duplication suggesting that ANG promotes cell proliferation by regulating chromosome replication together with activating the proliferative signaling pathway and enhancing ribosome biogenesis [2]. Interactions with proteins involved in the proteolysis and protein transport reflect other possible biological roles of ANG.

ANG can be internalized by receptor-mediated endocytosis [19]. The internalized ANG undergoes nuclear translocation and increases rRNA transcription, which is essential for cell proliferation [5], [7], [19]. Accumulating evidence has shown that ANG also localizes in the cytoplasm [1], [8], [9]. However, the role of cytosolic ANG is unknown. Our results demonstrated that ANG interacts with the three stress fiber components, i.e. ACTB, ACTN4, and MYH9. Co-localization analysis revealed that ANG interacts with these proteins at the leading edge of migrating cells. It should be noted that α-actin and ACTN2, which are smooth muscle specific isoforms of ACTB and ACTN4, have been reported to interact with ANG [17], [27]. Therefore, ANG might interact with different isoforms of cytoskeleton proteins in different types of cells. Down-regulation of ANG resulted in increased but less motile stress fibers (Figure 3,4), which is associated with enlarged but less dynamic FAs (Figure 5). All these data suggested that the cytosolic ANG plays an important role in stress fiber assembly and focal adhesion formation.

ACTB is a globular protein that polymerizes into long filaments (F-actin) [28]. ACTN4 is an actin crosslinking protein that links actin filaments together [29]. MYH9 forms the myosin motors that slide actin filaments past one another to make the fiber contract [30]. These three proteins are main components of stress fibers, which anchor to FAs and generate forces to move and reshape the cell [13]. By interacting with these proteins, ANG might ensure to form thinner but more dynamic stress fibers to accommodate the needs of cell migration. We have observed the increased assembly of stress fibers in ANG-deficient cells (Figure 3). A possible explanation for this increased assembly is that ANG binds to ACTN4 to prevent ACTN4-mediated cross-linking of actin bundles. Other studies have shown that ANG induces formation of unstructured aggregates of G-actins under low KCl concentrations, thereby inhibiting actin polymerization *in vitro* [18]. Therefore, ANG may also bind to ACTB (Figure 2) to suppress the formation of new stress fibers by blocking the polymerization of G-actin. Further studies are needed to fully elucidate the actions of ANG in this process, including mapping the binding sites on ANG, constructing ANG mutants that lack stress fiber-binding activities, and determining the influence of these ANG mutants on stress fiber formations. The ANG mutants would lose regulatory activities on stress fiber formation and cell migration if the interactions between ANG and stress fiber components are vital for these processes.

Stress fibers are contractile actin filament bundles that are typically associated at both their ends to focal adhesions. Strong stress fibers end in large FAs, while weak stress fibers provide small FAs [13]. Our data showed that the reduction of ANG led to the enlargement of FAs (Figure 5,7), which is consistent with the enhanced formation of stress fibers. Cells exhibit a biphasic migration velocity in response to increasing adhesion strength, since cell migration depends on both FA assembly and disassembly [31]. In other words, it is the dynamics of FAs that controls cell migration. FA dynamics parallels integrin activation and the downstream phosphorylations of FAK. Tyr-397 and Tyr-925 are the main phosphorylation sites of FAK during its activation. It was reported that integrin stimulates FAK phosphorylation at Tyr397 to create a high-affinity binding site for the Src-homolog 2 (SH2) domain of Src family kinase (SFK). This FAK-Src complex acts to control cell shape and focal contact turnover events during cell motility [32]. Activated Src thereafter phosphorylates FAK at Tyr925, which is involved in modulating focal contact dynamics in motile cells [24], [33]. Our data showed the phosphorylations of FAK at Tyr397 and Tyr925 were inhibited in ANG siRNA-treated cells (Figure 6A,7A), indicating that ANG is an essential factor in FAK activation. Consistently, we observed retarded cell migration in ANG-deficient cells (Figure 6,7).

Cell migration is a key process in both tumor angiogenesis and cancer cell metastasis. It has been demonstrated that ANG facilitates the migration of vascular cells such as endothelial cells [10], [11]. Here we provided evidence to support the hypothesis that ANG promotes cancer cell migration as well. Based on our findings, we suggest that the existence of ANG in the cytoplasm ensures proper stress fiber assembly and FA maturation, and maintains FAK activation and FA dynamics, thus guaranteeing cell migration. It should be noted that current finding that ANG promotes cell migration through regulating stress fiber assembly and focal adhesion dynamics are based on 2D cell culture experiments, which may not necessarily have the same structure and dynamics in-vivo. Further studies using mouse models are warranted to elucidate the roles of ANG on cell migration during tumor angiogenesis or cancer cell metastasis.

## Materials and Methods

### Cell culture

HeLa cervical carcinoma cells were obtained from ATCC and cultured in DMEM (Invitrogen, Camarillo, CA) supplemented with 10% fetal bovine serum (Hyclone, Logan, UT). HUVECs were obtained from Cascade Biologics (Portland, OR) and cultured in SFM (Invitrogen) and M199 (Hyclone) (9∶16) supplemented with 3 mg/mL ECGS (Millipore, Billerica, MA), and 10% fetal bovine serum (Invitrogen). Human skin fibroflasts (HSF) were obtained from the Lawrence Berkeley National Laboratory (Berkeley, CA) and cultured in α-MEM (Invitrogen) supplemented with 10% fetal bovine serum. Cells were maintained at 37°C with 5% humidified CO_2_.

### Antibodies

Goat anti-ANG antibody (R&D Biosystem, Minneapolis, MN) was used for co-immunoprecipitation experiments. The anti-ANG antibody used for immunoblotting was generated in New Zealand white rabbits and purified with protein A agarose. Mouse monoclonal anti-ANG antibody used for immunofluorescence was a gift from Dr. Guofu Hu (Department of Pathology, Harvard Medical School) [5]. The other antibodies were: anti-β-actin (Santa Cruz Biotechnology, Santa Cruz, CA), anti-α-actinin 4 (Aviva Systems Biology, San Diego, CA), anti-myosin heavy chain (Abcam, Cambridge, MA), anti-phospho-FAK Tyr397 (Cell Signaling Technology, Danvers, MA), anti-phospho-FAK Tyr925 (Cell Signaling Technology), anti-FAK (Cell Signaling Technology), and anti-paxillin (Millipore).

### Co-immunoprecipitation

Because HeLa cells express low level endogenous ANG, we treated the cells with 500 ng/mL exogenous ANG for 24 h to increase the intracellular amount of this protein. The cells treated with or without ANG were lysed with RIPA buffer (20 mM Tris-HCl, pH 7.5, 150 mM NaCl, 1 mM Na_2_EDTA, 1 mM EGTA, 1% NP-40, 1% sodium deoxycholate, 2.5 mM sodium pyrophosphate, 1 mM Na_3_VO_4_) with freshly-added complete protease inhibitor cocktail (Roche Applied Science, Indianapolis, IN). Cell lysates were incubated with affinity-purified goat anti-ANG antibody at 4°C overnight, and then precipitated with 30 µL protein G agarose (Millipore) for 1 h. After washing 3 times with a buffer (20 mM Tris-HCl, pH 7.5, 150 mM NaCl, 1% NP-40), immunocomplexes were boiled directly in loading buffer, and subjected to sodium dodecyl sulfate polyacrylamide gel electrophoresis (SDS-PAGE). To confirm the interactions, we used the similar co-immunoprecipitation assay with the IgG group as another control.

### Protein identification by mass spectrometry

Silver-stained SDS-PAGE gels were photographed, and the bands that differentiated between the ANG-treated group and the control were cut out, hydrolyzed with modified trypsin, and subjected to a tandem quadrupole-quadrupole-time-of-flight mass spectrometer (QqTOF QSTAR® Elite MS; Applied Biosystems, Foster City, CA) equipped with a high-performance liquid chromatography (HPLC; Michrom Bioresources, Inc., Auburn, CA) with a nanoelectrospray (ESI) head for maximal sensitivity. The MS data were used to search for matches in the nonredundant protein database at the National Center for Biotechnology Information (human-subset) (http://www.ncbi.nlm.nih.gov, released on 23 April 2008) using Protein Pilot™ 4.0 software (Applied Biosystems, Inc.). The threshold for protein identification was set at >95% confidence, and the precursor-ion mass tolerance and fragment-ion mass tolerance were set at ±0.1 Da. The results were then organized, and putative proteins were annotated using the Database for Annotation, Visualization, and Integrated Discovery (DAVID).

### Immunoblot analysis

Protein samples were separated by SDS-PAGE and then transferred to nitrocellulose membranes (Whatman, Clifton, NJ). Membranes were blocked with 3% bovine serum albumen in TBS-T buffer (20 mM Tris-HCl, pH 8.0, 150 mM NaCl, 0.05% Tween-20), and incubated with primary antibodies at 4°C overnight. After 3 washes with TBS-T, membranes were incubated with HRP-conjugated secondary antibodies, reacted with the SuperSignal West Pico chemiluminescence substrate (Pierce, Rockford, IL), and then exposed to X-ray film.

### RNA interference

RNA interference was carried out with designed siRNAs. ANG siRNA 1 was annealed with 5′-AAGAAUGGAAACCCUCACAGA-3′ (forward), and 5′-UCUCUGUGAGGGUUUCCAUUC-3′ (reverse) as described by Kishimoto *et al.* [20]; ANG siRNA2 was annealed with 5′-GCAUCAAGGCCAUCUGUGATT-3′ (forward), and 5′ -UCACAGAUGGCCUUGAUGCTG-3′ (reverse); and negative control siRNA was annealed with 5′-UUCUCCGAACGUGUCACGUTT-3′ (forward), and 5′-ACGUGACACGUUCGGAGAATT-3′ (reverse). All siRNAs were synthesized by GenePharma (Shanghai, China). siRNAs were transfected into HeLa cells and HUVECs with Lipofectamine 2000 (Invitrogen). Cells were further analyzed after 48 h transfection.

### Stable Transfection of HeLa Cells

ANG RNA interference plasmid (pBS/U6-ANGi) was a gift from Dr. Guofu Hu at Harvard Medical School. The target sequence was 5′-GGTTCAGAAACGTTGTTGTTA-3′. pBS/U6-ANGi or empty vector pBS/U6 were cotransfected with pBabe-puro into HeLa cells using Lipofectamine 2000 (Invitrogen) and the stable transfectants were selected with 1 µg/mL puromycin for 2 weeks.

### Immunofluorescence

Cells were fixed in 4% paraformaldehyde for 15 min at 4°C and permeated with 0.2% Triton X-100. After a blockade with goat serum for 1 h at room temperature, cells were incubated with primary antibodies (26-2F, anti-paxillin, anti-β-actin, anti-α-actinin 4, or anti-myosin heavy chain) for 1 h at room temperature. The incubation with secondary antibodies was then carried out at room temperature for 1 h in the dark. In the co-localization assay, two target proteins were sequentially stained. To stain F-actin, cells were incubated with rhodamine-phalloidin at room temperature for 1 h. A confocal microscope (LSM510 Meta, Carl Zeiss, Jena, Germany) was used for observation and imaging.

### Quantification of stress fibers and focal adhesions

The differences in stress fibers were quantified as previously described [21]. Briefly, ImageJ software was used to generate line profiles. A graphic depiction was then generated where the *x*-axis represented the distance across the cell, the *y*-axis represented the level of fluorescence, and each immunofluorescence intensity spike represented an individual stress fiber crossed by the line. To distinguish the true stress fibers from the background, we also drew several lines outside the cells and determined the intensities on the lines. The fluorescence level of one hundred was set as the cutoff since the fluorescence intensity (FI) outside the cells was never greater than this value. We randomly selected six cells and three regions in each cell for quantification. The FI was classified into two levels, low intensity (FI<1000), and high intensity (FI≥1000). The number of stress fibers at each level was quantified.

We randomly selected seven cells in each group to quantify the number and size of focal adhesions using ImageJ software [22]. A particle analysis was performed on images to select FAs based on anti-paxillin staining, and then the number and size of the particles were quantified.

### Time-lapse videomicroscopy

HeLa cells treated with ANG siRNA and control siRNA were seeded in 6-well plates, imaged with the confocal microscope for 8 h, and then manually tracked with ImageJ software. The direction of movement, distance and velocity were recorded.

When studying cytoskeleton dynamics in live cells, we transfected the cells with plasmids coding for red fluorescent protein (RFP)-tagged actin (RFP-actin, kindly provided by Prof. Bähler from Westfalian Wilhelms-University, Germany) together with control siRNA or ANG siRNA. Cell protrusions were imaged every two minutes under the time-lapse confocal microscope. The images were then made into movies using Windows Movie Maker.

### Wound healing assay

HeLa cells or HUVECs plated in 35-mm dishes were transfected with ANG siRNA or control siRNA. When cells grew to confluence, a line was traced with a 20 µL pipette tip. HeLa cells were then incubated with DMEM containing 10 ng/mL EGF. The wound was photographed at 0, 24, and, 48 h. HUVECs were incubated with serum-free medium, and the wound was photographed at 0, 4, and 8 h. To rescue the effect induced by ANG knockdown, exogenous ANG was added to the medium.

### Statistical analysis

All experiments were repeated at least three times. The data were expressed as the mean ± SD and evaluated with a double-sided Student's t test. Values of *P*<0.05 were accepted as statistically significant in any analysis.

## Supporting Information

Figure S1 **Exogenous ANG treatment did not affect the expression levels of stress fiber components.** HeLa cells were treated with or without ANG, and the mRNA levels of *MYH9* (A) *ACTN4* (B), and *ACTB* (C) were detected by RT-qPCR and normalized to *GAPDH* gene. The protein levels of MYH9, ACTN4, ACTB, GAPDH, and ANG were detected by immunoblot (D).
(TIF)

Figure S2 **The assembly of stress fibers increased in HeLa cells stably transfected with ANG interference plasmid.** HeLa cells were stably transfected with pBS/U6 or pBS/U6-ANGi plasmids. (A) The total RNAs were subjected to real-time quantitative PCR (left panel), and the cell lysates were immunoblotted with anti-ANG antibody (right panel). (B) Cells were fixed and stained with rhodamine-phalloidin.
(TIF)

Movie S1 **The dynamics of actin cytoskeleton decreased in ANG-deficient HeLa cells.** (A) The dynamics of actin cytoskeleton marked by RFP-actin in HeLa cells treated with control siRNA. (B) The dynamics of actin cytoskeleton marked by RFP-actin in HeLa cells treated with ANG siRNA. The process lasts 18 min in each cell.
(MP4)
