# Supplementary figures and images for: Angiogenin Enhances Cell Migration by Regulating Stress Fiber Assembly and Focal Adhesion Dynamics
Source: PLoS One. 2011 Dec 14;6(12):e28797. doi: 10.1371/journal.pone.0028797 (PMC3237552; doi:10.1371/journal.pone.0028797)

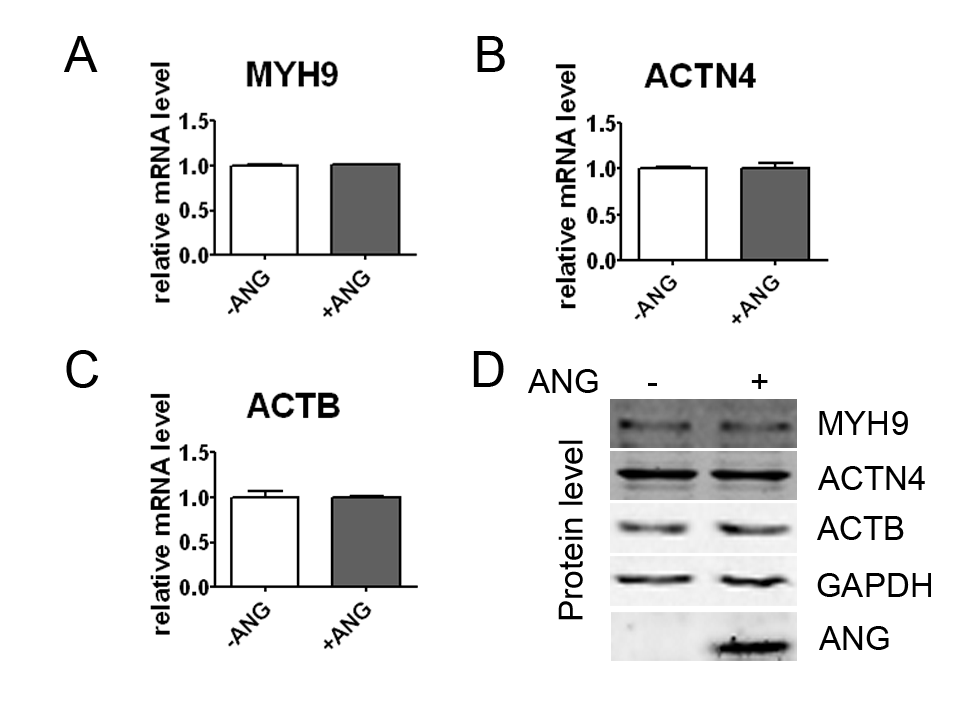

Supplement: Figure S1 — Exogenous ANG treatment did not affect the expression levels of stress fiber components. HeLa cells were treated with or without ANG, and the mRNA levels of MYH9 (A) ACTN4 (B), and ACTB (C) were detected by RT-qPCR and normalized to GAPDH gene. The protein levels of MYH9, ACTN4, ACTB, GAPDH, and ANG were detected by immunoblot (D). (TIF) [file pone.0028797.s001.tif]

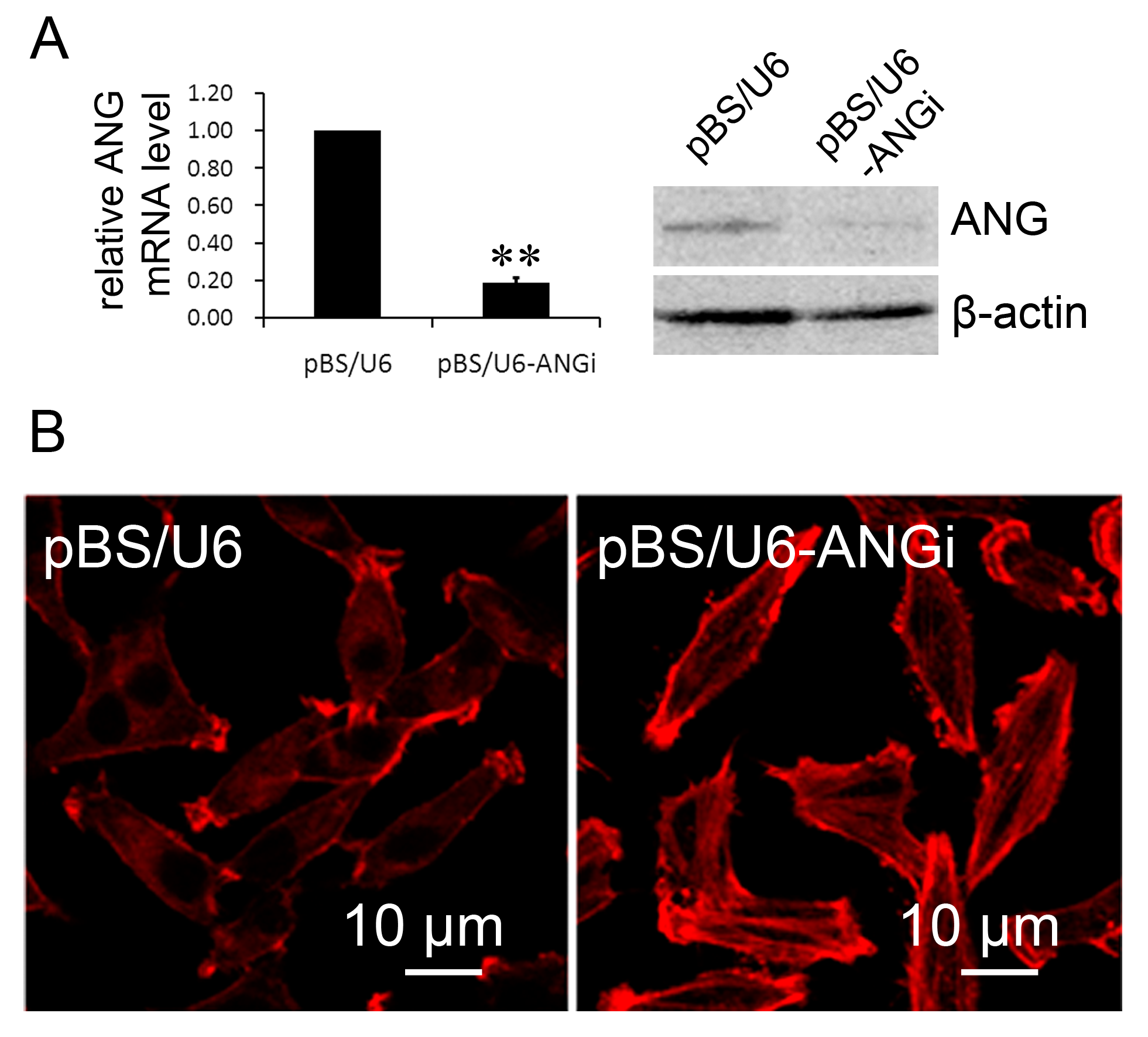

Supplement: Figure S2 — The assembly of stress fibers increased in HeLa cells stably transfected with ANG interference plasmid. HeLa cells were stably transfected with pBS/U6 or pBS/U6-ANGi plasmids. (A) The total RNAs were subjected to real-time quantitative PCR (left panel), and the cell lysates were immunoblotted with anti-ANG antibody (right panel). (B) Cells were fixed and stained with rhodamine-phalloidin. (TIF) [file pone.0028797.s002.tif]
